# Early home treatment of childhood fevers with ineffective antimalarials is deleterious in the outcome of severe malaria

**DOI:** 10.1186/1475-2875-7-143

**Published:** 2008-07-29

**Authors:** Adebola E Orimadegun, Olukemi K Amodu, Peter E Olumese, Olayemi O Omotade

**Affiliations:** 1 Institute of Child Health, College of Medicine, University of Ibadan, Ibadan, Nigeria; 2 Global Malaria Programme, World Health Organization, Geneva, Switzerland

## Abstract

**Background:**

Early diagnosis and prompt treatment including appropriate home-based treatment of malaria is a major strategy for malaria control. A major determinant of clinical outcome in case management is compliance and adherence to effective antimalarial regimen. Home-based malaria treatment with inappropriate medicines is ineffective and there is insufficient evidence on how this contributes to the outcome of severe malaria. This study evaluated the effects of pre-hospital antimalarial drugs use on the presentation and outcome of severe malaria in children in Ibadan, Nigeria.

**Methods:**

Two hundred and sixty-eight children with a median age of 30 months comprising 114 children with cerebral malaria and 154 with severe malarial anaemia (as defined by WHO) were prospectively enrolled. Data on socio-demographic data, treatments given at home, clinical course and outcome of admission were collected and analysed.

**Results:**

A total of 168 children had treatment with an antimalarial treatment at home before presenting at the hospital when there was no improvement. There were no significant differences in the haematocrit levels, parasite counts and nutritional status of the pre-hospital treated and untreated groups. The most commonly used antimalarial medicine was chloroquine. Treatment policy was revised to Artemesinin-based Combination Therapy (ACT) in 2005 as a response to unacceptable levels of therapeutic failures with chloroquine, however chloroquine use remains high. The risk of presenting as cerebral malaria was 1.63 times higher with pre-hospital use of chloroquine for treatment of malaria, with a four-fold increase in the risk of mortality. Controlling for other confounding factors including age and clinical severity, pre-hospital treatment with chloroquine was an independent predictor of mortality.

**Conclusion:**

This study showed that, home treatment with chloroquine significantly impacts on the outcome of severe malaria. This finding underscores the need for wide-scale monitoring to withdraw chloroquine from circulation in Nigeria and efforts intensified at promoting prompt treatment with effective medicines in the community.

## Background

Malaria remains a significant cause of morbidity and mortality among children and creates enormous social, economic and disease burdens in endemic regions[1,2]. Current control efforts focus on reducing malaria-attributable morbidity and mortality. Prompt evaluation of all febrile illness, case-recognition and use of appropriate antimalarial therapy are essential to malarial control in order to optimize clinical outcomes of malaria-infected patients.

In Africa, majority of malaria episodes occur in rural areas with considerable variation in the treatment seeking pattern with over 50% self treated at home [3-5]. Self-treatment with drugs bought from local private drug sellers has been widely reported widely, ranging from between 4–87% of cases [3,6,7]. Help from the public health services is usually sought only if the home based treatment is considered ineffective [8,9]. The main reasons for these delays are inaccessibility of the health services, limited time, transport or financial constraints to visit them [10]. Although mothers and care-givers are able to recognize symptoms associated with uncomplicated malaria, fewer than 15% of episodes are treated correctly [11]. Patients presenting with malaria often receive inappropriate drugs or in incorrect doses in spite of trainings given to shop keepers or care-givers in many communities [12]. Thus, pre-hospital use of antimalarial drugs may also confound some of the symptoms and signs that may be useful in assessment and appropriate management decisions in the hospital.

Drug resistance remains a major problem affecting progress on management and control of malaria [13,14]. Chloroquine (CQ) resistance has been associated with low drug accumulation [15]. The spread of decreasingly sensitive *Plasmodium falciparum *may be promoted by the presence of sub-curative concentrations of the antimalarial medicine in the blood, that will only eliminate the most sensitive parasites allowing those that are less sensitive to survive [16], thus providing a biological advantage to the parasite, allowing the spread of chloroquine resistant forms [17]. Based on the efficacy profile of CQ, ACT replaced CQ as the first line antimalarial medicine in Nigeria in 2005. The new recommended first-line antimalarial drug for home treatment is artemether-lumefantrine (AL). Following deregulation of AL to an over-the-counter (OTC) status, it is also the recommended option for community/home-based treatment of malaria. AL was distributed free for under-five children in many government hospitals, however the supply remains irregular. However in many areas, and even in government hospitals, CQ is still being prescribed because of the high cost and unavailability of AL. While many current programmes are promoting home treatment of malaria, studies in Nigeria have shown that antimalarials may be used inappropriately even when prescribed in the hospital [13,14].

Many studies have evaluated the effects of different antimalarial chemoprophylaxis in Nigerian children, [19-21] which resulted in severe morbidity in most cases and a higher prevalence of malaria in the specific study areas. However, the effects of inappropriate antimalarial taken early in the course of a febrile illness has not been documented. Due to lack of availability and other constraints, mothers and caregivers continue to use CQ in the initial treatment of febrile episodes at home before seeking additional care when there is no improvement. Therefore, this study was carried out to evaluate the effects of pre-hospitalization antimalarial drugs use on the morbidity and outcome of severe malaria cases in children admitted to the paediatric emergency units in Ibadan.

## Methods

The study was carried out in Ibadan, south-west Nigeria, a region that is exposed to stable, malaria transmission with seasonal [1]. A total number of 268 children presenting with fever, malaria parasitaemia and clinical features consistent with the WHO [22] criteria of severe malarial anaemia (SMA) and cerebral malaria (CM) (without co-morbidity, such as pneumonia or diarrhoea) were recruited consecutively at the Children's Emergency Ward and Children Out-Patient Clinic of the University College Hospital, Ibadan and the Adeoyo Maternity Hospital, Ibadan.

Ethical approval was obtained from the joint University of Ibadan, University College Hospital, Ibadan Ethical Committee, the Oyo State Ministry of Health and the WHO ethical review committee. Written informed consent was obtained from the parents or guardian of the patients prior to enrollment. A semi-structured questionnaire was used to collect data on patients' demographic characteristics, history of febrile illness, antimalarial drug treatments given during current episode of clinical malaria prior to presentation at the hospital and findings from physical examination findings at the time of admission. All patients had and clinical history and examination performed, followed by blood films taken from finger pricks. Malaria parasitaemia was confirmed with thick blood smears stained with Giemsa examined for trophozoites of *P. falciparum *independently by two trained and experienced microscopists. Patients were managed on admission according to WHO guidelines and National Antimalarial Policy.

Data was entered and analysed using SPSS 11.0 for Windows (SPSS Inc., Chicago, USA). Descriptive statistics (medians, ranges) were computed for continuous variables while frequencies were computed for categorical variables. Comparisons of continuous variables were computed using Mann-Whitney U test because of their non-parametric nature while comparisons of categorical variables were done using the Fishers exact test because of the small numbers in some subcategories. The logistic linear regression technique was used to investigate the predictive value of pre-hospital use of antimalarial and outcome (died vs. survived) after inclusion of age and clinical severity in the models. A *p*-value of < 0.05 was taken as being statistically significant.

## Results

The study patients comprised 155 (57.8%) male and 113 (42.2%) female giving a male to female ratio of 1.4:1. The age of the patients ranged from five months to 10 years with a median age of 30 months. One hundred and sixty eight (62.7%) of the study population had pre-hospital antimalarial treatments. Table 1 compared the age, haematocrit, parasite counts and nutritional status of patients who received and did not receive pre-hospital antimalarial treatments. The median age of patients who had pre-hospital treatment, 31.5 months was significantly higher than 25.5 months in those who did not receive antimalarial treatment (p = 0.013). There was no significant difference in the median haematocrit levels of the pre-hospital treated and untreated group (p = 0.297). Though a higher malaria parasite counts was found in the group of patients who did not receive pre-hospital antimalarial drugs but this did not differ significantly from the parasite counts estimate in the group who received pre-hospital treatments (p = 0.837).

**Table 1:** Comparisons of age, parasite counts and haematocrit

	Treatment given N = 168	Treatment not given N = 100	Mann-Whitney U	p
Age (months)				
Range	5–120	5–120	6875.0	0.013
Median	31.5	25.5		
Haematocrit (%)				
Range	5–34	6–35	7449.5	0.297
Median	18	16		
Parasite counts (/μl)				
Median	4137142	768000	8092.5	0.837
Nutritional status				
Range	-6.37–2.90	-8.95–1.91	7962.5	0.635
Median	-1.38	-1.25		

Of the 268 patients with severe malaria, 114 (42.5%) were cases of CM while 154 (57.5%) had SMA without coma (Table 2). A significantly higher proportion of CM (71.1%) than SMA (56.5%) patients had antimalarial drugs before presenting in the hospital (p = 0.016). There was no significant gender difference in the distribution of patients in respect of treatment given before presentation (p = 0.371). Although about two-thirds (61.9%) of the study patients came from the lower socioeconomic class family but there was no significant difference in the distribution of patients who had pre-hospital antimalarial treatment between upper and lower classes (p = 0.606).

**Table 2:** Characteristics of subjects

	Treatment Given		Treatment Not Given		RR(95% CI)	*P
			
	n	%	N	%		
Gender						
Male	101	65.2	54	34.8	1.10 (0.91, 1.33)	0.371
Female	67	59.3	46	40.7		
Diagnosis						
CM	81	71.1	33	28.9	1.26 (1.05, 1.51)	0.016
SMA	87	56.5	67	43.5		
Social Class						
Lower	102	61.4	64	38.6	0.95 (0.79, 1.15)	0.606
Upper	66	64.7	36	35.3		

CM – Cerebral malaria, SMA- Severe malarial anaemia, OR-Odds ratio

*Fishers exact test used

CQ was the most commonly used antimalarial drug accounting for 54.2% of the children who received antimalarial treatment. CQ was given to 49 (43.0%) and 42 (27.3%) of CM and SMA, respectively (p = 0.009). Amodiaquine was given to 12 (10.5%) of CM and 8 (5.2%) of SMA groups. Sulphadoxine-pyrimethamine was given to 7 (6.1%) of CM patients and six (3.9%) of SMA patients. Of the 168 children who received an antimalarial drug before admission, 48.2% had CM compared with 33% in the group of patient who had no antimalarial treatment (p = 0.015, RR = 1.26). Table 3 shows that 49 (53.8%) children out of 91 who had CQ treatment compared with 33 (33.0%) in the no treatment group developed CM. The risk of presenting as CM was 1.63 times higher if the child was treated with CQ at home. Table 4 shows the outcome of the severe malaria cases and compared the proportion of those who had pre-hospital antimalarial drugs among those that survived and those that died. The overall mortality rate of severe malaria was 6.7%. Two-thirds of the children who died had pre-hospital treatment with CQ. Table 5 shows the logistic regression model for potential predictors of death in severe malaria. Controlling for age and clinical severity (CM or SMA), pre-hospital treatment with CQ remained a significant predictor of death. The risk of death was four times higher if a child had been given CQ before presentation than if he had no pre-hospital treatment with CQ.

**Table 3:** Diagnoses by chloroquine use and no pre-hospital treatment given

Drug Given	Chloroquine		No treatment given		Total	
CM	49	53.8	33	33.0	82	42.9
SMA	42	46.2	67	67.0	109	57.1
Total	91	100.0	100	100.0	191	100.0

P = 0.005, RR = 1.63, 95% CI = 1.16, 2.29

Fishers exact test used

**Table 4:** Risk of death in severe malaria after pre-hospital antimalarial drugs

Antimalarial drugs	Died (18)		Survived (250)		RR(95% CI)	*p
			
	n	%	n	%		
Chloroquine	12	66.7	79	31.6	4.25(1.54–11.7)	0.004
Amodiaquine	2	11.1	17	6.8	1.71(0.36–8.07)	0.371
Sulfadoxine-pyrimethamine	1	5.6	11	4.4	1.33(0.16–10.93)	0.559
Quinine	1	5.6	0	0.0	-	0.067
Halofanthrin	1	5.6	2	0.8	7.29(0.62–84.5)	0.189
Artesunate only	1	5.6	1	0.4	14.6(0.87–24.5)	0.130

*Fishers exact test used

**Table 5:** Logistic regression model for potential predictors of outcomes in severe malaria

Factors	B	S.E	p	Exp(β)	95% CI
Chlorquine (given vs not given)	1.367	0.528	0.010	3.923	1.39, 11.05
Age (months)	0.008	0.012	0.512	1.008	0.98, 1.03
CM vs SMA	0.903	0.532	0.089	2.467	0.87, 6.99
Constant	-1.939	1.512	0.200	0.144	-

## Discussion

Early treatment with effective antimalarial treatment has been demonstrated to decrease the morbidity and mortality of malaria in endemic countries. In most countries in the sub-Saharan Africa however, there has been little success in reducing both infant and child mortality even with the focus on better management of malaria. The present study has shown that pre-hospital antimalarial treatment of febrile children remains a significant practice among child care-givers in Nigeria as previously reported from the same area [11]. The prevalence of CQ use, 54.2% in this present study, is high despite the recent National drug policy change from CQ to ACTs.

Pre-hospital administration of pre-packaged chloroquine was previously reported to have reduced the risk of developing severe malaria among children in some African communities at a time when CQ was still effective [23]. Our data has shown that pre-hospital administered CQ was associated with an increased risk of severe malaria, with a concurrent increase in mortality. This may be due to a recent upsurge in the incidence of CQ resistance malaria in Nigeria. This findings corroborates a recent report by Sowunmi and co-workers [24] that observed failure to clear parasites within three days and the prevalence of fever two days after commencement of CQ were strong indicators or predictors of malaria treatment failure in Nigerian children.

However, in a previous report from the same centre, CQ resistance of *P. falciparum *in vitro was significantly higher in isolates from patients with severe malaria than those with uncomplicated disease [13]. The clinical implication of these findings was that severe malaria patients were less likely to respond to CQ than cases of uncomplicated malaria and this association was thought to be due to either progression of uncomplicated to severe disease following CQ failure or increased virulence of CQ resistant parasites. In the light of the findings from this study, CQ resistance was a determinant of malaria severity and mortality which may be due to the selection of increasingly virulent CQ resistant forms promoted by the presence of sub-curative concentrations of the antimalarial drug in the blood that will only eliminate the most sensitive parasites allowing those that are less sensitive to survive [17].

A recent study from Nigeria has shown that correct use of chloroquine increased from 2.6% to 52.3% among home care-givers after training, however as high as 47.7% still wrongly treat febrile children [12]. This finding underscores the need to review the present policy on home management of malaria. If this is not adequately addressed, even the use of more efficacious antimalarials like the ACTs, still poses a risk to effective home based management of malaria at the community level.

In addition, there is some evidence to suggest that households of a high social class are more likely to use antimalarials to which there is less parasite resistance. As majority of our patients were from lower socioeconomic class, reducing the cost, improving the availability and access to quality pre-packaged artemisinin-based antimalarial may improve pre-hospital treatment of malaria and thus prevent resistance [25]. White [26] observed that the main obstacles to the success of combination treatment in preventing the emergence and spread of resistance, is incomplete or inadequate treatment. Thus, factors such as availability, wide coverage of good quality drugs and adequate antimalarial treatment regimens need to taken into cognizance to prevent emergence of resistance to ACTs. Furthermore, early diagnosis and prompt treatment are major strategies for malaria control. In the light of our findings, early treatment with ineffective medicines can result in worse outcome.

In conclusion, the use of ineffective antimalarial medicines for the home-based management of malaria could be a hindrance to prompt access to effective treatment, as the caregivers delay seeking treatment convinced they have initiated antimalarial treatment, albeit with compromised medicines. This study has demonstrated this practise to be deleterious on the outcome of malaria. To maximise the impact of chemotherapy on malaria morbidity and mortality, resources are urgently required to facilitate the use of ACTs for home-based treatment of malaria.

## Competing interests

The authors declare that they have no competing interests.

## Authors' contributions

AEO and OKA contributed equally in the conception, design, analysis of the data and drafted the manuscript. PEO participated in the interpretation of the data and helped in drafting and writing up the manuscript. OOO participated in the design of the study and contributed to drafting and writing up the manuscript. All the authors read and approved the final manuscript.
